# Functionalised graphene sheets as effective high dielectric constant fillers

**DOI:** 10.1186/1556-276X-6-508

**Published:** 2011-08-25

**Authors:** Laura J Romasanta, Marianella Hernández, Miguel A López-Manchado, Raquel Verdejo

**Affiliations:** 1 Instituto de Ciencia y Tecnología de Polímeros, ICTP-CSIC, Juan de la Cierva 3, 28006, Madrid, Spain

**Keywords:** dielectric properties, graphene, interfacial polarisation, nanocomposites, silicones

## Abstract

A new functionalised graphene sheet (FGS) filled poly(dimethyl)siloxane insulator nanocomposite has been developed with high dielectric constant, making it well suited for applications in flexible electronics. The dielectric permittivity increased tenfold at 10 Hz and 2 wt.% FGS, while preserving low dielectric losses and good mechanical properties. The presence of functional groups on the graphene sheet surface improved the compatibility nanofiller/polymer at the interface, reducing the polarisation process. This study demonstrates that functionalised graphene sheets are ideal nanofillers for the development of new polymer composites with high dielectric constant values.

PACS: 78.20.Ci, 72.80.Tm, 62.23.Kn

## Introduction

In recent years, elastomeric materials with high dielectric constant have been considered for different functional applications such as artificial muscles, high charge-storage capacitors and high-K gate dielectric for flexible electronics [1,2]. Several methods have been explored in order to increase their dielectric permittivity although the most common approach involves the addition of high dielectric constant ceramics to the elastomeric matrix. This strategy usually requires high loading fractions and, hence, produces an unwanted increase of the system rigidity for the applications already mentioned [3-5]. In some other cases, dielectric constant increments have been met with relatively high loss tangent values (tg (*δ*)) and frequency dependence which is also undesirable for capacitor applications [6,7]. Obtaining composites with both high dielectric permittivity and low loss tangent values at the same time is specially challenging due the interfacial polarisation or Maxwell-Wagner-Sillars (MWS) process. This mechanism occurs at the interface between materials with different permittivities and/or conductivities and involves rather high *ε*' and tg (*δ*) values at low frequencies due to the accumulation of virtual charges at the filler/polymer interface [8]. Altering the interfacial interaction between filler and polymer matrix can regulate the dielectric contrast between matrix and filler and thus, prevent the MWS polarisation [9-11]. Therefore, chemical modification of filler particles has to be taken into account in order to achieve high permittivity composites with low dielectric losses. Nevertheless, filler surface modifications can significantly raise the production costs and thus, make them unfeasible to be produced on large scale.

Thermally expanded graphene sheets are of great interest to overcome the aforementioned problems. The thermal reduction of the graphite oxide has the advantage to produce chemically modified graphene sheets (or so-called functionalised graphene sheets FGS) without the need of further modification steps. Besides, the huge aspect ratio of these carbon-based nanoparticles (experimental value 1850 m^2 ^g^-1^) [12] reduces considerably the percolation threshold compared to any other type of high dielectric constant filler. Accordingly, very small loading fractions can offer interesting permittivity enhancements without adversely affecting the dielectric losses and mechanical properties of a given polymer matrix.

In this work, as-produced carbon nanotubes (CNTs) and thermally expanded graphene sheets are compared for their possible enhancing effect on an elastomer dielectric response. Results show that FGS are an ideal candidate as high dielectric constant fillers in capacitor applications. The presence of remaining functional groups at their surface is able to improve the filler-matrix compatibility, enhance the nanoparticle distribution and make them suitable to develop novel, flexible and easy to process capacitors with relatively high dielectric constant and low tg (*δ*) values.

## Experimental

A commercial poly(dimethyl)siloxane (PDMS) kindly supplied by BlueStar Silicones (Rhodorsil MF620U) was used as elastomeric matrix.

Both CNTs and FGS employed in this study were synthesised in our laboratories as follows: aligned multi-wall CNTs were produced by chemical vapour deposition (CVD) injection method using toluene as the carbon source and ferrocene as the catalyst. A 3 wt.% ferrocene/toluene solution was injected into a hot quartz tube reactor (760°C) at 5 ml h^-1 ^under inert atmosphere. FGS were produced by reduction and thermal exfoliation of graphite oxide (GO). GO was previously produced using natural graphite powder (purum powder ≤ 0.1 mm, Fluka, Sigma-Aldrich Corp. St. Louis, MO, USA) according to the Brödie method [13]. Rapid heating (30 s at 1,000°C) of the graphite oxide under inert atmosphere produced the partial thermal decomposition of the functional groups (epoxy, hydroxyl and carboxyl groups) present in the GO, splitting the GO into FGS through the evolution of CO_2 _(gas). Both CNT and FGS were used without further treatments.

Nanocomposites containing 0.5, 1.0, and 2.0 wt.% of CNT and FGS were prepared at room temperature in an open two-roll laboratory mill (speed ratio of 1:1.4) using standard mixing procedures. After that, samples were vulcanised at 170°C in an electrically heated hydraulic press using the optimum cure time (*t*_90_), deduced from the curing curves previously determined by means of a rubber process analyser (RPA2000 Alpha Technologies, Akron, OH, USA).

Broadband dielectric spectroscopy was performed on an ALPHA high-resolution dielectric analyser (Novocontrol Technologies GmbH, Hundsangen, Germany). Cross-linked film disc-shaped samples were held in the dielectric cell between two parallel gold-plated electrodes. The thickness of the films (around 100 μm) was taken as the distance between the electrodes and determined using a micrometre gauge. The dielectric response of each sample was assessed by measuring the complex permittivity ε*(ω) = ε'(ω) - jε"(ω) over a frequency range window of 10^1 ^to 10^7 ^Hz at 23°C. The amplitude of the alternating current (ac) electric signal applied to the samples was 1 V. In this work, the real part of the complex permittivity constant will be referred simply as the dielectric permittivity constant.

Stress-strain measurements were performed on a tensile test machine (Instron 3366 dynamometer, Norwood, MA, USA) at 23°C. Dog bone-shaped specimens with thickness around 0.5 mm were mechanically cut out from the vulcanised samples. The tests were carried out at a crosshead speed of 200 mm min^-1 ^with a distance between clamps of 2.0 mm. The elongation during each test was determined by optical measurement (video extensometer) of the displacement of two marker points placed along the waist of the tensile test sample. An average of five measurements for each sample was recorded.

Nitrogen-fractured cross-sections of the composites were examined by scanning electron microscopy (SEM), (ESEM XL30 Model, Philips, Amsterdam, Netherlands). Samples were sputter-coated with a thin layer of 3 to 4 nm of gold/palladium lead prior to imaging.

## Results and discussion

### Dielectric properties

**Figure 1:**
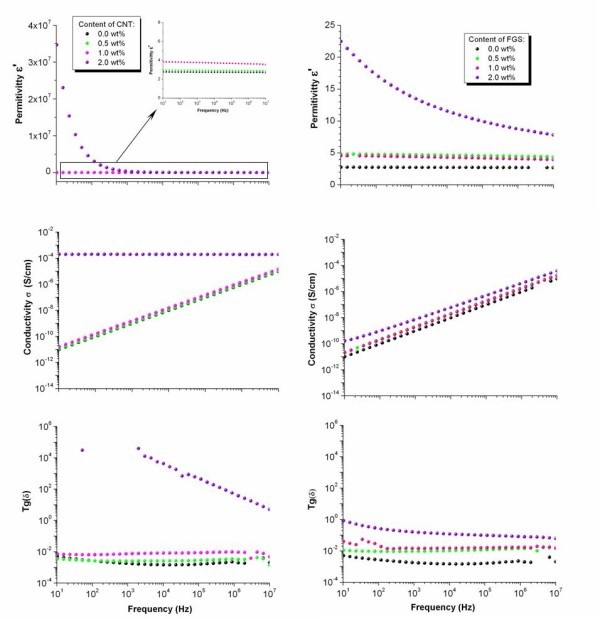
**Dielectric permittivity, conductivity (σ) and loss tangent (tg (*δ*)) as a function of frequency**. These were measured at room temperature, for (left) CNT/PDMS and (right) FGS/PDMS composites at various filler concentrations.

**Figure 2:**
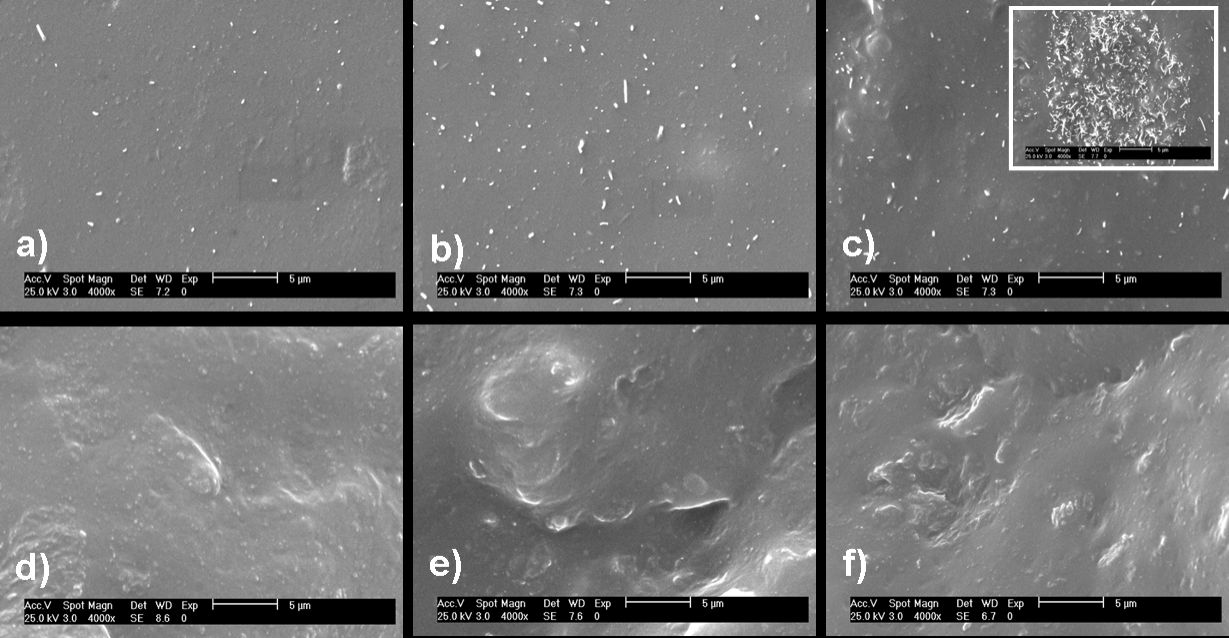
**SEM images of CNT/PDMS and FGS/PDMS composites**. (Top) SEM images of CNT/PDMS composites: (**a**) 0.5 wt.%, (**b**) 1.0 wt.%, and (**c**) 2.0 wt.%. The inset shows CNT agglomerates present in the sample. (Bottom) SEM images of FGS/PDMS composites: (**d**) 0.5 wt.%, (**e**) 1.0 wt.%, and (**f**) 2.0 wt.%. The scale bar corresponds to 5 μm.

The dielectric properties of the poly(dimethyl)siloxane (PDMS) matrix and composites with different CNT and FGS contents, measured at room temperature are shown in Figure 1. The permittivity constant was significantly increased by the addition of both carbon nanoparticles in the whole frequency range. While the dielectric permittivity of the composite with 0.5 wt.% of CNT (*ε' *= 2.9) did not substantially differ from that of the neat elastomer (*ε' *= 2.7), the sample containing 1.0 wt.% of CNT showed an electrical insulator behaviour with a permittivity constant increase of 1.5 times (*ε' *= 4.0). Hence, the electronic charge for composites up to 1.0 wt.% of CNT remained confined on isolated carbon nanotubes by the insulating polymer matrix (see Figure 2a, b). Meanwhile the composite with 2.0 wt.% of CNT showed a dielectric permittivity increase of six orders of magnitude. This abrupt increase in the permittivity value is ascribed to the motion of free charge carriers due to the formation of a continuous conductive pathway throughout the medium between CNT clusters (see Figure 2c). For this composite, the large increase in the loss tangent as a function of the frequency shows the existence of a strong interfacial polarisation phenomenon, clearly indicating that CNT/PDMS composites are percolative systems with a critical weight fraction between 1.0 and 2.0 wt.% of CNT. On the other hand, the dielectric permittivity spectra for composites with only 0.5 to 1.0 wt.% of FGS were characterised by a smooth and frequency-independent behaviour, with values about two times higher than that of the PDMS matrix in the whole frequency range. For composites with 2.0 wt.% of FGS, the value of the permittivity constant raised up to *ε' *= 23 towards low frequencies, which is ten times over the pure matrix. Although the conductivity spectrum of this composite showed an insulating character, the increase in the dielectric permittivity as the frequency decreases suggests that ion accumulation at the graphene/polymer interface starts to appear. Nevertheless, the loss tangent value hardly varies over all the frequency range, which can be attributed to: (1) the homogenous dispersion of FGS in the elastomeric matrix (see Figure 2d, e, f) and, (2) the presence of the functional groups on the graphene sheet surface, which interrupts the π-conjugation in the graphene layers, diminishes the surface electrical conductivity and favours the filler/polymer compatibility [14].

Figures of Merit (FoM) are widely used to compare composites with modified properties. In order to describe the relative enhancement of the dielectric permittivity in a given polymer matrix with respect to the weight fraction (*w_2_*) of the filler employed, a FoM can be defined as follows [15]:

(1) $$FoM=\frac{\left(\frac{\varepsilon \prime_{c}-\varepsilon \prime_{1}}{\varepsilon \prime_{1}}\right)}{w_{2}}$$

Where $\varepsilon_{c}^{\prime }$ and $\varepsilon_{1}^{\prime }$ are the composite and polymer matrix dielectric permittivity, respectively. For comparison, several examples of PDMS composites with different fillers have been taken from the literature (see Table 1). In all cases, the values selected correspond to the lowest amount of filler with the highest permittivity enhancement possible, that is, the composite sample with filler concentration below the percolation threshold. As it can be observed, the FoM for our composites containing FGS is 1 or even 2 orders of magnitude higher than the rest of the cases. The impact of the FGS on broadband dielectric permittivity is very high compared to the low weight fraction used.

**Table 1:** FoM calculated for several types of high dielectric constant filler/silicone composites

Filler	Filler loading (wt.%)	FoM
TiO_2 _[3]	70.0	3.33 (at 1 Hz)
TiO_2 _[4]	30.0	1.11 (at 10 Hz)
PMN-PT [3]	70.0	2.38 (at 1 Hz)
BaTiO_3 _[3]	70.0	8.09 (at 1 Hz)
PHT [20]	1.0	21.42 (at 10 Hz)
CuPc [21]	20.0	5.0 (at 1 kHz)
CNT*	0.5	14.8 (at 10 Hz)
FGS*	0.5	157.77 (at 10 Hz)
FGS*	2.0	366.29 (at 10 Hz)

*Values reported in the present work

### Mechanical behaviour

The influence of the carbon-based nanoparticles on the mechanical properties is shown in Table 2. The addition of either CNTs or FGS resulted in a slight decrease of the elongation at break values although a good stretchability was retained. Both types of carbon-based nanoparticles also produced a slight increment in the stiffness of the composites, being this effect more pronounced for samples with FGS, which is also an indication of improved adhesion between FGS and the polymer matrix. Several studies in literature focusing on the mechanical properties of graphene-filled polymer nanocomposites also revealed an increase in modulus as a function of loading fractions, being the larger improvements in elastomeric matrices due to their lower intrinsic modulus as recently pointed out in several reviews about graphene/polymer nanocomposites [16,17]. The results here presented agree with a comparative study of both FGS and CNT in an epoxy resin carried out by Rafiee et al. [18]. These authors also showed greater improvements for FGS than for CNT/polymer systems and suggested that the reason for this enhanced adhesion could be the wrinkled topology of thermally expanded graphene, mainly caused by the defects produced either during graphite oxidation or graphite oxide thermal exfoliation. This nanoscale roughness together with the high specific surface area and the two-dimensional geometry could result in improved mechanical interlocking and adhesion with polymeric chains [18,19].

**Table 2:** Stress at several strains and elongation at break for silicone and its composites

Filler content (wt.%)		Stress at 100% strain (MPa)	Stress at 300% strain (MPa)	Stress at 500% strain (MPa)	Elongation at break (%)
	0.0	0.33 ± 0.05	0.71 ± 0.09	1.49 ± 0.18	842 ± 23
CNT	0.5	0.43 ± 0.05	0.83 ± 0.10	1.69 ± 0.22	754 ± 45
	1.0	0.74 ± 0.18	1.38 ± 0.30	2.76 ± 0.59	732 ± 38
	2.0	0.69 ± 0.06	1.35 ± 0.15	2.67 ± 0.39	583 ± 14
					
FGS	0.5	0.57 ± 0.03	1.33 ± 0.07	2.75 ± 0.19	651 ± 18
	1.0	0.54 ± 0.08	1.29 ± 0.21	2.46 ± 0.43	644 ± 39
	2.0	0.99 ± 0.03	2.15 ± 0.09	3.38 ± 0.17	528 ± 32

## Conclusions

The electrical properties of CNT and FGS fillers on a silicone elastomeric matrix were studied for their possible enhancing effect on the material dielectric response. The increase on the dielectric permittivity depended on the filler content and frequency; although, FGS had a larger effect on the dielectric permittivity without significantly altering the tg (*δ*) value. An increase in the permittivity value, about 10 times higher than that of PDMS, was obtained at low frequency for composites with 2.0 wt.% of FGS. The presence of functional groups on the graphenes' surface and their homogenous dispersion throughout the polymer matrix was effective enough to modify the dielectric characteristics of the interface, increasing the dielectric permittivity value without the introduction of loss mechanisms. The addition of both filler nanoparticles caused a slight increment in the elastic modulus at different strains, being this fact more evident for composites containing FGS. The wrinkled morphology and the high specific surface area of the FGS employed resulted in improved adhesion with the polymeric chains. A slight decrease of elongation at break values was observed for both types of composites although good stretchability was retained.

The homogeneous FGS/silicone nanocomposites prepared in this study display desirable mechanical and dielectric properties, indicating potential applications in the electronic industry.

## Abbreviations

CNTs: carbon nanotubes; CVD: chemical vapour deposition; FGS: functionalised graphene sheets; FoM: Figures of Merit; GO: graphite oxide; MWS: Maxwell-Wagner-Sillars; PDMS: poly(dimethyl)siloxane; SEM: scanning electron microscopy.

## Competing interests

The authors declare that they have no competing interests.

## Authors' contributions

LJR carried out the synthesis and characterisation of both nanofillers and nanocomposites, participated in the discussion and drafted the manuscript. MH performed the dielectric analysis, participated in their theoretical interpretation and helped to draft the manuscript. MALM helped in nanocomposite preparation, participated in the discussion and revised the manuscript. RV designed and coordinated the study, led the discussion of the results and revised the manuscript. All the authors read and approved the final manuscript.
